# Multivariate analysis of long-term climate data in connection with yield, earliness and the problem of global warming

**DOI:** 10.18699/vjgb-24-18

**Published:** 2024-04

**Authors:** V.М. Efimov, D.V. Rechkin, N.P. Goncharov

**Affiliations:** Institute of Cytology and Genetics of the Siberian Branch of the Russian Academy of Sciences, Novosibirsk, Russia; Institute of Cytology and Genetics of the Siberian Branch of the Russian Academy of Sciences, Novosibirsk, Russia; Institute of Cytology and Genetics of the Siberian Branch of the Russian Academy of Sciences, Novosibirsk, Russia

**Keywords:** climate, global warming, models, next generation breeding, adaptability, earliness, климат, глобальное потепление, модели, селекция будущего, адаптивность, скороспелость

## Abstract

Climate change is the key challenge to agriculture in the XXI century. Future agricultural techniques in the Russian Federation should involve the optimization of land utilization. This optimization should apply algorithms for smart farming and take into consideration possible climate variations. Due to timely risk assessment, this approach would increase profitability and production sustainability of agricultural products without extra expenditures. Also, we should ground farming optimization not on available empirical data encompassing limited time intervals (month, year) or human personal evaluations but on the integral analysis of long-term information bodies using artificial intelligence. This article presents the results of a multivariate analysis of meteorological extremes which caused crop failures in Eastern and Western Europe in last 2600 years according to chronicle data and paleoreconstructions as well as reconstructions of heliophysical data for the last 9000 years. This information leads us to the conclusion that the current global warming will last for some time. However, subsequent climate changes may go in any direction. And cooling is more likely than warming; thus, we should be prepared to any scenario. Plant breeding can play a key role in solving food security problems connected with climate changes. Possible measures to adapt plant industry to the ongoing and expected climate changes are discussed. It is concluded that future breeding should be based on the use of highly adapted crops that have already been produced in pre-breeding programs, ready to meet future challenges caused by potential climate change.

## Abstract (ru)

Изменение климата – определяющая проблема растениеводства XXI в. Агротехнологии будущего в Российской Федерации должны быть связаны с оптимизацией землепользования, которая будет осуществляться как с применением алгоритмов «умного» сельского хозяйства, так и на основе прогноза потенциальных климатических изменений. Это позволит повысить рентабельность и устойчивость производства сельскохозяйственной продукции без дополнительных затрат за счет своевременного учета возможных рисков. При этом оптимизация системы возделывания культур должна базироваться не на имеющихся эмпирических показателях, полученных по ограниченным во времени точкам учета (месяц, год), и субъективной человеческой оценке, а на комплексном анализе массивов многолетней информации. В настоящей работе приведены результаты многомерного анализа метеорологических экстремумов и связанных с ними неурожаев в Восточной и Западной Европе за последние 2600 лет по летописным данным и палеореконструкциям, а также реконструкциям гелиофизических данных за последние 9000 лет. Отмечается, что идущее глобальное потепление продлится еще некоторое время. Однако последующие изменения климата могут быть направлены в любую сторону, причем, скорее, в сторону похолодания, а не потепления, поэтому надо быть готовыми к любым сценариям будущего. Для нивелирования последствий этих изменений селекция может сыграть ключевую роль в решении проблем продовольственной безопасности. Обсуждаются перспективы разработки мер адаптации растениеводства к происходящим и ожидаемым изменениям климата, и сделан вывод, что селекция будущего должна базироваться на использовании уже отработанных в программах предварительной селекции (pre-breeding) высокоадаптированных сельскохозяйственных культур, потенциально отвечающих будущим вызовам, обусловленным потенциальным изменением климата.

## Introduction

Geologic record indicates that dramatic climate changes directly affected humanity throughout its history (Gupta, 2004). The cooling in the Palearctic, sometimes reaching extreme values, started in the late Pleistocene, about 27 ka BP, and ended about 14 ka BP (Prentice, 2009). Archeological data show that humans inhabiting the Palearctic either successfully adapted to the changes or migrated to areas with better conditions. In VIII–X centuries BC, the latter group proceeded to productive economy (Shnirelman, 2012), that is, farming based on plant domestication and, later, animal domestication (Goncharov, 2013). At present, it is obvious that Toynbee’s (1954) ‘challenge and response’ theory of the history of civilisations, which presumes that the transition to agriculture was the response of hunters and gatherers to abrupt aridization caused by the melting of Late Pleistocene glaciers, has found no evidence (Trifonov, Karakhanyan, 2004). Moreover, agriculture in West Asia started against the background of wears relative humidization.

Local and global climatic changes constitute the primary concern for the XXI century, particularly, its first decades, when measures for mitigation of severe consequences for humanity and the global agroecosystem are urgent (Eckardt et al., 2023). The oncoming climate change may exert numerous adverse effects on crop production throughout the globe. It will demand significant extension of biodiversity (germ plasm pool), required for the inclusion of new characters and traits and completely new previously uncultivated plant species in breeding. The collection, preservation, and subsequent effective and intelligent use of the biodiversity of crops and their wild relatives in the changing climate are critical issues (Eastwood et al., 2022).

Many relatives of commercially important crops have considerable polymorphisms for seasonal adaptation (Goncharov, Chikida, 1995; Leigh et al., 2022; Liang, Tian, 2023); thus, they can be useful for improving the general adaptability of crops to local and global climate changes.

The consequences of climate changes cause anxiety to experts in various fields (Kattsov et al., 2011; Ruddiman et al., 2016), including biologists and agrarians, who deal with a wide variety of objects (Baltzoi et al., 2015; Gurova, Osipova, 2018; Morgounov et al., 2018; Eastwood et al., 2022; and others). It is beyond doubt that current climatic trends adversely influence the performance of many extensively grown crops. These trends are a considerable commercial risk to global agriculture and other farm industries (Lobell, Gourdji, 2012). It is predicted that climate changes will affect not only the production but also the quality of food (Atkinson et al., 2008), thereby jeopardizing food security. The broadening of polymorphism for many characters (Trifonova et al., 2021) with ensuing breeding for optimal duration of vegetative period (earliness) of crops are becoming more and more relevant (Kamran et al., 2014; Smolenskaya et al., 2022).

The potential climate changes demand modification of breeding programs for new generation cultivars and breeds. The requirements for higher adaptability to future environmental condition changes should be put at the forefront, which will apparently differ from the present. Neither the scale nor the directions of the changes can be unambiguously assessed. Present forecasts concern mainly risks associated with the broad (standard) use of conventional crop architectonics (Jatayev et al., 2020; Liu et al., 2022). This problem is significant, because about 70 % of dwarf common wheat commercial cultivars bear only two alleles of the Rht genes – Rht-B1b and/or Rht-D1b (Sukhikh et al., 2021).

There is no consensus among investigators as to whether the recorded climate changes are caused by anthropogenic or natural factors (Ruddiman et al., 2016; Lobkovsky et al., 2022; and others). At last time, the Sun is shown to operate in distinct modes – a main general mode, a Grand minimum mode corresponding to an inactive Sun, and a possible Grand maximum mode corresponding to an unusually active Sun (Solanki et al., 2004; Usoskin et al., 2014). It states that the heat flux from the Sun to Earth, the so-called solar constant, is in fact not constant, at least, on the millennial scale. This flux demonstrates variations unpredictable with the current state of knowledge

It is clear that the presently observed global warming started long before the industrial boom. We just live in a time of changes, when the solar heat flux on our planet starts its change once again to induce a climatic upheaval (Usoskin et al., 2014; Biswas et al., 2023). In view of all this, the analysis of historical and modern data to assess the possible limits of natural climatic variations appears to be essential for choosing land use strategies and for successful farming in the future.

The goal of this study is to analyze the limits of climate variability, meteorological extremes, and crop failures in Eastern and Western Europe over 2600 years from chronicles (Barash, 1989), paleoreconstructions of air temperatures (Sleptsov, Klimenko, 2005), and composite solar physics data across millennia based on proxy methods (Clette et al., 2014; Wu et al., 2018). We applied multivariate analysis approaches, in particular, the principal component analysis (PCA), for more comprehensive coverage and deeper analysis of the processes under study.

## Materials and methods

The following data were invoked:

1) chronicle data on years with meteorological extremes and crop failures in Western and Eastern Europe over 2600 years, from X century BC to XVI century AD from monograph of S.I. Barash (1989);

2) climate reconstructions for Eastern Europe (East European Plain) over the last 2000 years according to paleoclimatic data reported by A.M. Sleptsov and V.V. Klimenko (2005);

3) Solar activity reconstruction over the last 9000 years according to indirect data (Wu et al., 2018);

4) Wolf sunspot numbers SN(v2.0) (1700–2022) from (WDC-SILSO, Royal Observatory of Belgium, Brussels).

**Fig. 1.**
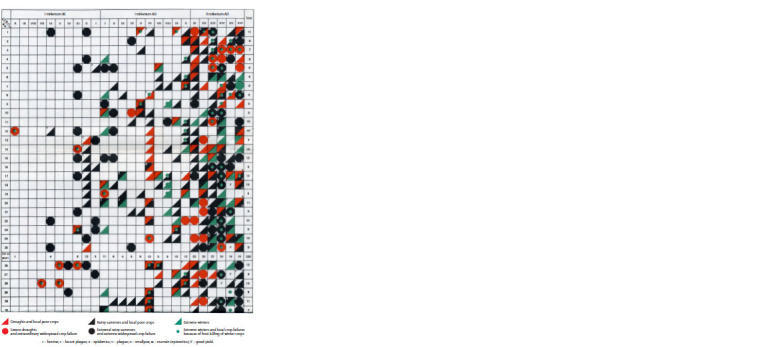
Meteorological extremes and crop failures in Western (WE) and Eastern (EE) Europe over 2600 years, from X century BC to XV century AD, fragment from (Barash, 1989).

We added some attributes to chronicle data presented in S.I. Barash (1989) to compensate insufficient accuracy in the characterization of years. Specifically, we introduced an integral attribute bulking crop failures in general in addition to attributes reflecting data on failures caused by particular factors: drought, overflooding, etc. In this case, the formation of this characteristic was carried out exclusively according to the data of S.I. Barash (Fig. 1)

Long-term data were processed by the principal component analysis (PCA) for time series, PCA-TS (Karhunen, 1947; Loève, 1948). By this method, any time series can be expanded into principal components that reflect the trend, quasicyclic fluctuations, and noise (Efimov et al., 1988). The modern PCA-TS version (Efimov et al., 2021) converts a unidimensional time series into a trajectory matrix (Takens, 1981). The matrix of Euclidean distances between its rows is calculated, and principal components (PCs) are extracted from the latter by the master coordinate procedure (Gower, 1966). Time series intervals uniform in variability patterns are detected from phase images drawn from principal components.

It should be mentioned that the opinion that statistical independence of principal components presumes their functional independence and, therefore, no principal component can be another component’s derivative in the strict mathematical sense, because their correlation is zero, is false. A counterexample is the pair of time series, Sin(t) and Cos(t). The derivative of sine is cosine, their phase image is a circle, and their correlation is zero.

Similar situations often arise in the processing of real series by PCA. When one of the components is interpreted as a derivative of another, this fact can be used for predictions. Components often appear in pairs with close variances and frequencies, their phase images are close to circles, and which of them is the derivative of the other can be determined from the contributions of attributes to the components or from their shift with reference to each other. In another frequent case, contributions to one of the components are of the same sign and are similar in amplitude, forming a trend, whereas the other component is constituted by two sequential intervals of opposite signs, characterizing trend changes.

As each line has two directions, the researcher chooses the orientation at his discretion. Any principal component can be multiplied by “–1”. This will invert signs of contributions of all attributes. It is recommended that component orientation be chosen so that the signs of all contributions be positive in case of trend, whereas in case of opposite directions, negative contributions should be first and then positive ones. With this choice, the phase trajectory rotates mainly clockwise, a positive value of the derivative points to the growth of the principal component, and a negative, to its drop.


Supplementary Materials are available in the online version of the paper: https://vavilov.elpub.ru/jour/manager/files/Suppl_Efimov_Engl_28_2.pdf


The calculation of pairwise cross-correlations between the manifestations of the listed attributes of years involved the cutoff of noise related to less significant (minor) principal components (Supplementary Material 1)1. We confined our studies to the effects of the detected modulation on events mentioned in chronicles; therefore, the diagonal matrix elements in the table presented in Supplementary Material 2 are always below unity. The mean error of the correlation coefficient for the specified number of objects (n = 2600) and the mean correlation coefficient value (r = 0.500) is sr = (1 – r2)/√n no more than 0.014. Thus, pairwise correlation coefficients exceeding the triple mean error (0.044) were considered significant.

## Results

We analyzed the collected by S.I. Barash’s (1989) data transformed into matrix (see Fig. 1), in which objects are years and attributes in cells indicate favorable (good yield) or unfavorable (all other) events.

**Fig. 2.**
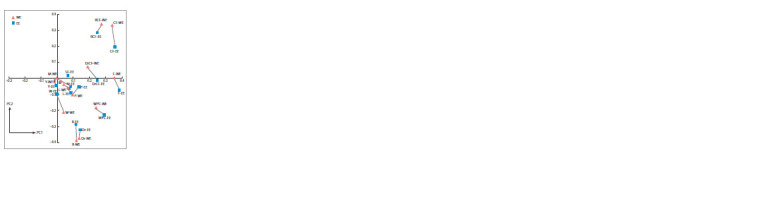
The similarity in the manifestation of year natural phenomena in Western (WE) and Eastern (EE) Europe with respect to PC1 and PC2.

Designations in what follows: Eastern Europe: Dr-EE, drought and local poor crops; DrCF-EE, severe drought, extreme widespread crop failure; R-EE, rainy summer, local poor crops; RCF-EE, wet summer and extreme widespread crop failure; W-EE, severe winter; WPC-EE, extreme winter and local poor crops because of frost-killing of winter crops; F-EE, famine; L-EE, locust plague; Epi-EE, epidemics; P-EE, plague; Sp-EE, smallpox; SF-EE, spot fever; M-EE, murrain (epizootics); Y-EE, good yield; CFEE, widespread crop failure. Western Europe: the same with the WE after the hyphen

We introduced the CF-EE and CF-WE attributes. In S.I. Barash (1989), lean years are not marked by the CF-EE and CF-WE, but are “coded” in a hidden way in the Dr, DrCF, R, RCF, and WPC. We resigned out their effect in the two CF-EE and CF-WE attributes and, after that, we assessed the influence of all the listed natural phenomena. We found that crop failures are affected only by the DrCF and RCF, associated with widespread crop failures, rather than by local poor crops.

The data presented by S.I. Barash (1989) lack any information on typhus epidemics in Western Europe; thus, the SF-WE is null. For this reason, the overall matrix shows 15 natural phenomena for Eastern Europe and 14 ones for Western, 29 in total.

The data were processed by the PCA (Fig. 2, Supplementary Materials 1–3). The first (PC1) and second (PC2) principal components account for 22 % of the total sample variance (see Supplementary Material 1). Eigenvalues are conventionally arranged in decreasing order. They reflect information redistribution and the concentration of the most significant factors in the principal components (see Supplementary Material 3).

The sum of all eigenvalues equals the dimensionality of the correlation matrix (the number of attributes in the original sample). Therefore, when no regularities in the interactions of attributes can be recognized, each eigenvalue should be unity in case of a correlation matrix calculated from the original data matrix with centered and normalized attributes. Therefore, value “1” can be considered the threshold whose crossing by a principal component reflects a factor essential for sample description. Correspondingly, principal components whose eigenvalues fall short of this threshold should be considered insignificant. We will follow the terminology used by experts in computer and radio sciences (Oppenheim, Schafer, 1975) and name the informative set of PCs signal and other, insignificant components, statistical noise (see Supplementary Material 1). Here we regard the major principal components PC1 to PC12, accounting for about 66 % of the overall variance of the sample in question, as signal. Their eigenvectors are shown in Supplementary Material 3.

The first principal component (PC1; 14.5 % of the overall variance) reflects the influence of climatic (natural) factors on widespread crop failures and, consequently, on famine in Eastern and Western Europe. The greatest contribution amplitudes are made by the attributes DrCF-EE, RCF-EE, WPC-EE, DrCF-WE, RCF-WE, and WPC-WE. Consequently, the contributions of associated attributes CF-EE, F-EE, CF-WE, and F-EE are also high. The nature of the factor can be defined as yield vs. famine.

The second principal component (PC2; 7.5 % of the overall variance) reflects the influence of the factor determined by the set of attributes Dr-EE, R-EE, RCF-EE, and WPC-EE and their counterparts Dr-WE, R-WE, RCFWE, W-EE, and CF-WE. In Western Europe, the signs of contributions of Dr-WE, R-WE, and W-EE are opposite to those of RCF-EE and CF-EE. Obviously, the secondrank factor in Western Europe shows that excess moisture in summer is the main cause of crop failures. In contrast, such climatic conditions in Eastern Europe do not result in crop failures, although they exert a certain influence on agriculture in general. The nature of the factor responsible for PC2 can be defined as differences in moisture regimes between Eastern and Western Europe.

By analyzing chronicle data on meteorological extremes and crop failures in Western and Eastern Europe for 2600 years, we assessed the similarities between the series of these events from the location of attributes in the phase space of principal components (see Fig. 2; Supplementary Material 2). For clarity, attributes similar in Eastern and Western Europe are connected with lines (see Fig. 2).

The third principal component (PC3; 5.8 % of the overall variance) shows that the effect of widespread crop failures in Western Europe manifests itself as a threat of famine, whereas in Eastern Europe famines are not so great threat as epidemics, first of all, plague. This fact stems from seeking food by the population of steppe and forest-steppe regions and contacts of humans with small animals inhabiting steppes and conveying plague: marmots, ground squirrels, tarbagans, and such.

**Fig. 3.**
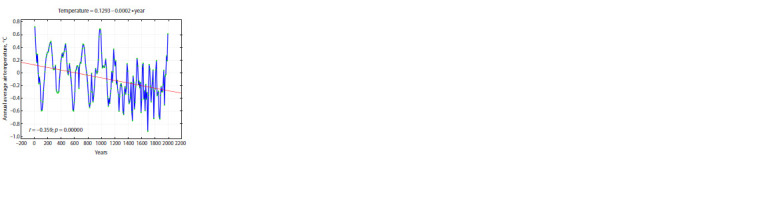
Annual temperature deviations from the present values in the East European Plain (averaged over decades). Data from (Sleptsov, Klimenko, 2005; Fig. 3).

We assessed climate changes on the grounds of data reported by A.M. Sleptsov and V.V. Klimenko (2005), who attempted to reconstruct climate in Eastern Europe (East European Plain) from four kinds of sources: instrumental measurements, historical evidence, palynology, and dendrochronology. They reconstructed the variation in the annual average air temperatures in the East European Plain for the last 2000 years (Fig. 3).

A.M. Sleptsov and V.V. Klimenko note a negative trend in annual average temperatures, most pronounced in the last millennium; more precisely, from year 1200 to the second half of the XX century. They extrapolate these data to the 50 years to come and conclude that the so-called “global warming” is in fact of anthropogenic nature and that it rescued humanity from a “global cooling”, which would have been much more disastrous for our civilization, as shown by the history of the XIV–XVIII centuries. Also, they reveal a clear climatic rhythm of about 200 years, closely associated with solar activity variation (Sleptsov, Klimenko, 2005).

**Fig. 4.**
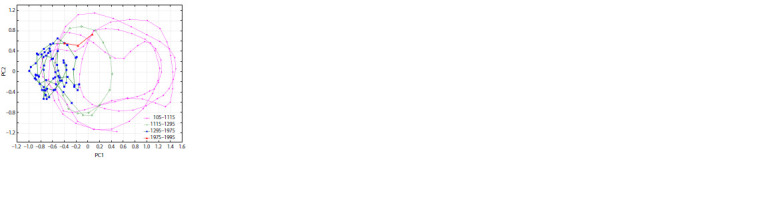
Phase image of the variation of annual average temperature in the East Siberian Plain on the plane of two major principal components, PC1 and PC2.

**Fig. 5.**
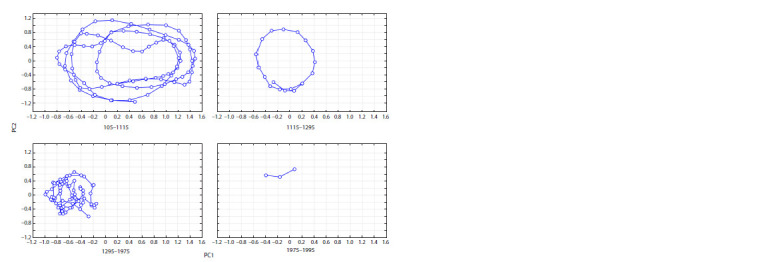
Phase images of the variation of annual average temperature in the East Siberian Plain on the plane of PC1 and PC2, separately for each temperature phase

**Fig. 6.**
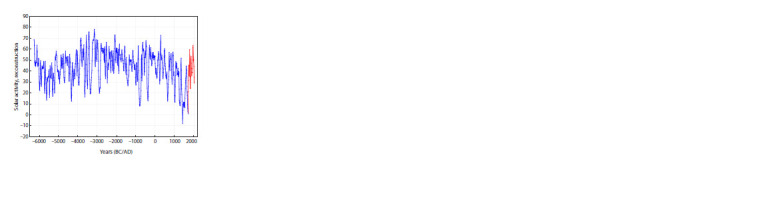
Solar activity reconstruction for the last 9000 years (Wu et al., 2018).

**Fig. 7.**
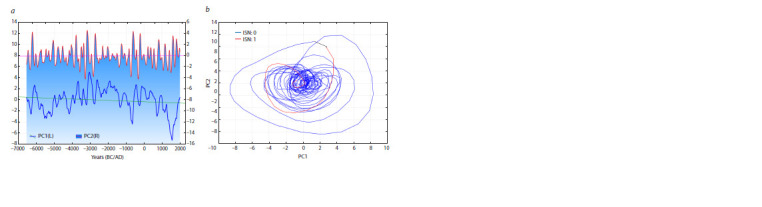
The first two principal components (PC1 and PC2) of solar activity over the last 9000 years (a), their phase image (b). Variances PC1 = 36.3 %, PC2 = 28.5 %.

A relatively beneficial time span lasted from the I to the XII century. After the XII century, it gave way to a cooling, which lasted nearly till present. However, additional information can be extracted from the data illustrated in Fig. 3. The processing of this time series by the PCA (Figs. 4–5) clearly demonstrates its nonuniformity. It can be concluded from the coefficients of correlation of the first two principal components (PC1 and PC2) with the annual average temperature with various lags that PC1 (47.7 % of variance) is responsible for air warming, and PC2 (24.9 % of variance), for its derivative (see the Table). This means that, when the trajectory of the series is above zero for PC2, it is bound to move right, towards higher temperatures, until it falls below zero and turns back. This regularity shows no significant deviations (Figs. 6, 7). Four phases with different regimes are recognized in the time span analyzed: cyclic oscillations with period about 200 years (years 105–1115), transitional phase (1115–1295); quasichaotic variation (1295–1975), and warming (1975–present), see Figs. 4 and 5.

**Table 1.**
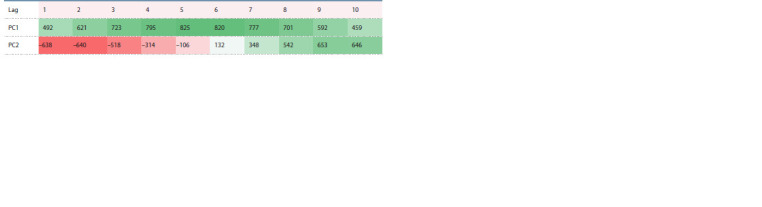
Correlation coefficients (×1000) of two major principal components with the annual average temperature in the East Siberian Plain with different lags Colors indicate: light-red and light-green – p <0.001; red and green – p <10–4.

It follows from the phase images in Figs. 6, 7 that (1) the trajectory of the time series under consideration has exceeded the limits that confined it in the last seven centuries, (2) it has not exceeded the limits in which it stayed in the entire I millennium and the beginning of the II millennium, and (3) it is not inconceivable that the cyclic regime characteristic of the I millennium is returning. If this conclusion is true, further temperature increase should be expected in the next 50–60 years for natural causes, not related to human activity.

The results of time series processing (see Figs. 4–6) confirm the inferences from our earlier analysis (Efimov, Goncharov, 2013). Specifically, climate in Western and Eastern Europe experiences centuries-long oscillations for presently unknown reasons, abruptly turning from one climate regime to another. The most notable transitions occurred in the I and II millennia AD and in the XIV– XVIII centuries. The climate leaps observed now (see Figs. 6, 7) may be indicative of another transition from one climate phase to the next, whose closest analogue is the regime of the I millennium, warmer and more arid than the present.

In addition, multivariate analysis allows recognition of clearly different time ranges of the influence of heat flow onto the Earth (see Fig. 7). The reconstruction of solar activity for the last 9000 years (see Fig. 7, a) brings us to the suggestion that it is undergoing a profound change in the form of heat flow onto the Earth (Wu et al., 2018). The start of this change should be dated back to the XVI–XVII centuries, thereby rejecting the hypothesis of industrial activity as the main cause of the present climate change. Figure 7, a shows that the I millennium AD was warmer than the preceding I millennium BC or the subsequent II millennium AD.

## Discussion

Our analysis of data reported by S.I. Barash (1989) indicates that lean years in Eastern Europe are those with droughts or excessive rains covering areas commensurable with the entire subcontinent (see Fig. 2, Supplementary Material 2). The same is observed in Western Europe, where the correlation between famine and excessive precipitation is more pronounced. Severe winters in Eastern Europe cause famine more often than in Western Europe. These differences stem from geographic features. Western Europe lies southwest and northeast and forms a natural barrier in the way of the North Atlantic Current, the main source of additional heat and moisture from the Atlantic (Hendry, 1982; Hogg, 1992; Hogg, Johns, 1995). The influence of Arctic air masses in Western Europe is weaker than in Eastern. In contrast, the effect of the North Atlantic Current on Eastern Europe is much weaker, and cold Arctic air masses greatly influence plant growth. Winter crops occupy a notable portion of arable areas in Eastern Europe, and their overwintering is of greater importance there.

The correlation between plague epidemics and crop failures in Eastern Europe is nearly two times closer than in Western (see Supplementary Material 2). This fact may be related to the predominance of arid and steppelike agrolandscapes in Eastern Europe. They are home to populations of steppe rodents, which are plague seeders. Just these animals closely approach human dwellings in times of food shortage. The situation with smallpox is different: it shows poor correlation in both Eastern and Western Europe. Smallpox virus is transmitted from human to human without animal vectors. Therefore, only intense human traveling matters in this case.

For unknown natural causes, climate in Western and Eastern Europe undergoes centuries-long changes with abrupt transitions from one regime to another (see Fig. 3). The most likely cause of these changes is millennia-long variations in heat flow from the Sun to the Earth. The most notable transitions were noted in the onsets of the I and II millennia (AD) and in the XIV–XVIII centuries. The climatic change observed at present may be a transition to another, unknown by now, climatic regime or the continuation of the cold climatic phase that started half a millennium ago. In fact, the current global warming began in the middle of the ХХ century, but it was considered the return to normal climatic conditions after the extraordinary cooling, the Little Ice Age of the XIV–XVIII centuries. It was not until recently that the notion appeared that this warming, if continued, would bring about catastrophic consequences, and humanity should be prepared for them in advance. As the main cause of this change was claimed to be human activity, its natural consequence was the illusion that it was the power of humanity to modify climate.

Although climate regime variations have long been studied and are of practical significance, their primary cause is still debatable. Some scientists state that they are caused by industrial activity (and proponents of this viewpoint succeeded in getting three Nobel awards: Peace Prize (Solomon et al., 2007), Prize in Economic Sciences (Nordhaus, 2019), and Prize in Physics (Manabe, 2019, 2023)). Others interpret the changes as a regular round of natural climatic fluctuations (Usoskin et al., 2014; Lobkovsky et al., 2022; and others).

The fact that the Earth receives nearly all heat from the Sun poses the question of regularities in the variation of this heat flow and predictability of changes. Naturally, attention is focused primarily on the trend and cyclic mode of the variation. However, as seen from analyses of solar activity, the results clearly depend on the scale of consideration. If we confine ourselves the epoch of regular direct solar activity observations over the last 300 years, the commonly known 11-year cycles are most pronounced. If we smooth them, the ascending trend is beyond dispute, and only unlimited increase can be forecasted, as is the present case

When we increase the scale to the last millennium, we see a Middle Age dip in the middle of the II millennium AD, out of which we are just coming. The only prediction in this case is further rise. In covering three millennia, we see that such dips happened before, but they were not as deep as the current; therefore, the temperature after the end of such a dip slowly drifts to cooling, being accompanied by minor fluctuations. The prediction will be a short-time rise followed by a gentle trend to cooling. If we analyze the information on the longest attainable time span (see Fig. 6), we see that big dips happened even during the greatest rises, e. g., on the cusp of the IV and III millennia BC. Such a change may happen in XXI century. The cause of such dips is unknown, and no reliable statistical regularities have been revealed.

## Conclusion

Scientists have long been discussing the prospects of using climatic models in the development of measures for the adaptation of various human activities to the current and expected climate changes (Kattsov et al., 2011, and others). Climate is fully responsible for what lives and grows in a certain biome. Lately, the effect of climate changes on farming has been extensively investigated (Rauner, 1981; Sirotenko, 2001; Zolotokrylin et al., 2020; Cooper, Messina, 2023; and others). However, assessments of agricultural response in various regions are diverse. The main cause of this fact is differences in source data, methods for data processing, and methods for evaluating the influence

The paradigm shift determined by the warming prediction is most often discussed in the context of probable aridization of huge areas (Trifonov, Karakhanyan, 2004), the resulting necessity of raising drought resistance of various crops (Zotova et al., 2020; Cooper, Messina, 2023), and search for new drought-resistant plant species applicable for cultivation (Baltzoi et al., 2015). Strategies of adaptation to climate changes may include better fitness of plant phenology to moisture availability (Ceccarelli et al., 2010), broader access to varieties with different duration of vegetative period (earliness) (Smolenskaya, Goncharov, 2023) in order to avoid stress at critical stages of their life cycles, water use improvement, and switch to breeding new-generation varieties for mitigating the rising unpredictability (Ceccarelli et al., 2010). Anyhow, breeders should take into consideration the high probability of climate changes in the decades to come, even if the formerly recorded extreme levels, which may aridize broad areas and shift agricultural zones from south to north, are not reached. In this case, the development of early varieties as a precautionary measure for improving agrocenosis adaptivity is an urgent task.

## Conflict of interest

The authors declare no conflict of interest.
